# Quantification of Hydrogen Peroxide in Cretan Honey and Correlation with Physicochemical Parameters

**DOI:** 10.1155/2021/5554305

**Published:** 2021-04-26

**Authors:** Amalia Alygizou, Spyros Grigorakis, Panagiota Gotsiou, Sofia Loupassaki, Antony C. Calokerinos

**Affiliations:** ^1^Department of Food Quality & Chemistry of Natural Products, Mediterranean Agronomic Institute of Chania (M.A.I.Ch.), Centre International de Hautes Etudes Agronomiques Méditerranéennes, P.O. Box 85, Chania 73100, Greece; ^2^Department of Chemistry, School of Physical Science, National and Kapodistrian University of Athens, Panepistimiopolis, Athens 15771, Greece

## Abstract

The aim of the present study is to quantify hydrogen peroxide, generated from various types of honey produced in Crete, as a potent antimicrobial agent, and establish any correlation with their physicochemical parameters. The basic physicochemical parameters (diastase activity, HMF content, moisture, electrical conductivity, color, and sugars) of 30 authentic honey samples were determined. The concentration of hydrogen peroxide in all samples was found to be within the range 0.010–0.092 mM. The known correlation between the electrical conductivity and the color of honey was confirmed in this study. Univariate and multivariate statistics applied to the results indicate that the results can be used to discriminate honey sample groups of different botanical origins.

## 1. Introduction

Honey besides its antioxidant, anti-inflammatory, and antimutagenic effects is also widely known for its antibacterial properties. It has been recorded as a medicine from ancient times due to wound-healing properties. There are several mechanisms responsible for the antibacterial properties of honey. Hydrogen peroxide is produced by the *Apis mellifera* (honeybee) glucose oxidase (GO) enzyme during dilution of honey, and it is produced in low but effective concentrations. Due to the slow release of hydrogen peroxide, there is much less cytotoxic damage to the cells of the patient, providing a more effective method than applying hydrogen peroxide directly to wounds [1].

Glucose oxidase (GOX) is most active in diluted or unripe honey, and when the sugar concentration is within 25–30% (w/w), hydrogen peroxide is produced:

(1) $$\begin{array}{c} \beta -\text{D}-\text{Glucose}+{\text{H}}_{2}\text{O}+{\text{O}}_{2}+\text{GO}⟶\text{gluconic acid}+{\text{H}}_{2}{\text{O}}_{2} \end{array}$$

Prolonged storage of honey reduces peroxide accumulation due to reduction of enzyme activity [2].

The evaluation of the endogenous hydrogen peroxide levels in honey can be of great value to predict the hydrogen peroxide-dependent antibacterial activity of honey and also to characterize or select honey samples for their use as an antibacterial agent or natural food preservative.

Environmental conditions can affect the physiology of the floral species or bee-related characteristics such as age or colony health, which might further affect the production of glucose oxidase [3].

Furthermore, accumulation of hydrogen peroxide of honey is affected by the content of glucose oxidase which appears to be formed during ripening. It is also affected by various minor components (nectar, pollen, and yeasts). The peroxide accumulation value of the honey also depends on the presence of high vitamin C content, handling, storage, and processing of honey. Moreover, pollen-derived catalase effectively hydrolyzes hydrogen peroxide to oxygen and water and is considered as a potent blocker of hydrogen peroxide accumulation [4–6].

Other studies have shown that the diversity in floral resources can have a direct effect on immune and bacterial factors and therefore on the glucose oxidase. Therefore, the level of hydrogen peroxide is the outcome of a dynamic equilibrium between the rate of its production and the rate of its destruction [7, 8].

The rate of hydrogen peroxide production also depends on dilution of honey. Bang et al. [9] reported that the maximum accumulation of hydrogen peroxide was achieved at 30%–50% (v/v) aqueous honey solutions. This can be explained by a factor that a certain degree of honey dilution facilitates access of the GOX to its substrate (glucose) and prevents GOX inhibition due to milieu acidification [10]. Moreover, apart from the glucose-glucose oxidase system, the auto-oxidation of polyphenols and flavonoids could degrade or destroy hydrogen peroxide. As reported by Brudzynski et al. [11] at low content, polyphenols in honey interact with hydrogen peroxide in the metal-catalyzed Fenton reaction to confer the oxidation action of hydrogen peroxide via generation of the hydroxyl radical, which is responsible for the oxidative damage to DNA caused by honey rather than molecular hydrogen peroxide [12].

All the above factors can, therefore, affect the hydrogen peroxide concentration of honey. Possibly nectar-derived peroxidases rather than catalase might be a cause of variation in the hydrogen peroxide-neutralizing capacity of different honey [1], and this has to be further studied in Cretan honey with relatively lower hydrogen peroxide production.

Since Crete is a major producer of honey in Greece but the most common quality characteristics have not been studied in detail, it was decided (a) to determine diastase activity, HMF content, moisture, electrical conductivity, color, and sugars in four different botanical groups (thyme honey, PDO “Pefkothymaromelo Kritis” honey (blend thyme-pine honey), pine honey, and Citrus honey), (b) to determine the amount of hydrogen peroxide produced after 30% (w/v) dilution with water, and (c) to establish any correlation between results. Although hydrogen peroxide in honey can be determined by using techniques like spectrophotometry, spectrofluorimetry, electrochemistry, chromatography, and chemiluminescence [13–17], in this work, it was decided to apply a hydrogen peroxide/peroxidase assay. To our knowledge, this is the first time that hydrogen peroxide in honey samples from Crete is quantified.

## 2. Materials and Methods

### 2.1. Materials and Chemicals

The following chemicals were supplied from Sigma-Aldrich Co. (St. Louis, MO, USA): hydrochloric acid, ammonium hydroxide (28–30% v/v), ethanol (95% v/v), sodium nitrate (≥98% w/w), sulfuric acid (20% v/v), sodium thiosulfate solution (0.10 N), sodium acetate buffer pH 5.30, potassium hexacyanoferrate(II) trihydrate, sodium acetate trihydrate (≥98.5% w/w), zinc acetate dehydrate (≥98% w/w), sodium bisulfate, sodium metabisulfite (≥97.0% w/w), D-(−)-fructose, sucrose, D-(+)-glucose, peroxidase from horseradish type II, and o-dianisidine (peroxidase substrate). From Merck (Germany), the following were obtained: potassium iodide for analysis EMSURE® ISO, Reagent Ph Eur, starch (soluble guaranteed reagent for analysis), phosphate buffer pH = 6.50, and hydrogen peroxide (30% v/v). Sodium chloride was supplied from Penta (Czech Republic), glycerol standard reference from Hanna Instruments (Gunstock, Rhode Island, USA), and acetonitrile Chromasolv™, for HPLC, gradient grade (≥99.9%) from Honeywell, Riedel-de Haën.

Ultrapure water of HPLC grade from an ultrapure water purification system with resistance of 12–18 *μ*Ω-cm was used throughout.

### 2.2. Apparatus

An HPLC chromatograph (Agilent 1100) with sample loop 20 *μ*l was used with column Lichrospher 100-NH2, 250 × 4 mm i.d., 5 *μ*m (Merck 1.50834), precolumn Lichrospher 100-NH2, 5 *μ*m (Merck 50958), and refractive index detector (Shodex RI-71, Japan).

All spectrophotometric measurements were made with a UV-visible diode array spectrophotometer (UvLine9400, Schott Instruments, USA).

A Conductivity Pocket Meter (Cond330i WTW, Germany) with cell (TetraCon 325/S, WTW, Germany), an AR 200 Automatic Digital Refractometer (Leica, Germany), and a Honey Color Photometer (HI 96785, Hanna Instruments, USA) were also used.

### 2.3. Honey Samples

Thirty honey samples of four botanical groups (thyme honey, PDO “Pefkothymaromelo Kritis” honey (blend thyme-pine honey), pine honey, and Citrus honey) were collected from different regions of Crete, coded, and stored at −20°C until analysis.

### 2.4. Methods

#### 2.4.1. Moisture Content

The moisture content (*W*) of honey was determined by a digital refractometer according to the method by the International Honey Commission [18], and calculations were made by using the following equation:

(2) $$\begin{array}{c} W=\frac{1.73190-\log\left(\text{RI}\right)}{0.002243}, \end{array}$$

where *W* is the water content in g per 100 g of honey and RI is the refractive index.

#### 2.4.2. Electrical Conductivity

10 g of dry sample was dissolved in 50.0 mL of deionized water. After complete mixing, the electrodes of the digital conductivity meter were inserted into the solution and the electrical conductivity (*S*_*H*_ in mS/cm abbreviated as EC) was calculated by the following formula [18]:

(3) $$\begin{array}{c} S_{H}=K\times G, \end{array}$$

where *K* is the cell constant (cm^−1^) and *G* is the conductance (mS).

#### 2.4.3. Hydroxymethylfurfural Content

Hydroxymethylfurfural content is determined after White according to the Harmonized Methods of the International Honey Commission [18]. More specifically, Carrez I solution (15.0 g of potassium hexacyanoferrate(II) dissolved in deionized water and diluted with deionized water to 100 mL) and Carrez II solution (30.0 g of zinc acetate dissolved in deionized water and diluted with deionized water to 100 mL) were prepared.

5.00 g of honey was mixed with 25 mL of deionized water and 0.5 mL Carrez I solution. After mixing the solutions, 0.5 mL of Carrez II solution was added and the mixture was diluted with deionized water to 50.00 mL. Filter the solution and transfer 5.00 mL of filtrate into each of two test tubes. Into one test tube transfer 5.00 mL of deionized water for the analyte measurement and into the other test tube transfer 5.00 ml of 0.20% w/v sodium bisulfite for the reference measurement. Measure the absorbance of the sample against the reference at 284 and 336 nm. The HMF content is calculated by using the following equation:

(4) $$\begin{array}{c} \text{HMF}\left(\text{mg}/\text{kg}\text{ of honey}\right)=\left({\text{Abs}}_{284}-{\text{Abs}}_{336}\right)\times 149.7\times 5\times D/W, \end{array}$$

where Abs_284_ and Abs_336_ are the absorbances at 284 and 336 nm, respectively, 149.7 and 5 are constants, *D* is the dilution factor if dilution of the sample is necessary, and *W* is the weight of the honey sample (g).

#### 2.4.4. Diastase Activity

Diastase activity is determined by using the Schade method according to the Harmonized Methods of the International Honey Commission [18].

#### 2.4.5. Honey Color Analysis

The color of the studied honey samples is analyzed with a ΗΑΝΝΑ Honey Color Photometer. The homogenous honey samples free from air bubbles are transferred into the cuvette (10 mm) which is introduced into the photometer. Color grades are compared to the glycerol standard and expressed in Pfund grades (mm).

#### 2.4.6. Determination of Hydrogen Peroxide

The concentration of hydrogen peroxide was enzymatically determined as described by White [19] and modified by Kwakman et al. [20]. The method is based on the reaction of hydrogen peroxide with *o*-dianisidine in the presence of horseradish peroxidase type II to form a colored product (brown color). Oxidized *o*-dianisidine reacts with sulfuric acid to form a more stable colored product (pink color). The intensity of the pink color measured at 540 nm is proportional to the original glucose concentration. For this analysis, 30% (w/v) water honey solutions were used. More specifically, 10 g of honey samples was dissolved in 5 mL of buffer (0.4 M pH = 6.50) and diluted on water until 25 mL. Then, the honey solutions were filtered through Whatman paper twice after which 120 mL of honey samples was added to 400 mL of peroxide reagent consisting of 50 *μ*g/mL *o*-dianisidine and 40 *μ*g/mL of horseradish peroxidase type II. The peroxide reagent was freshly prepared by mixing 5 mL of phosphate buffer (0.4 M, pH = 6.50) and 10 mg of *o*-dianisidine diluted to 2 mL of ethanol and then diluted further with water to 200 mL. The samples were incubated for 5 min at room temperature and stopped by the addition of 360 *μ*l of 6 M H_2_SO_4_. The absorption was measured at 540 nm. Moreover, in order to quantify the amount of hydrogen peroxide accumulated in diluted honey, a calibration curve of 30% (v/v) H_2_O_2_ dissolved to the concentration of 0.005–0.1 mM was used. The measurements were performed in triplicate for each sample. Results were expressed as mM of hydrogen peroxide in 30% (v/v) honey solution.

#### 2.4.7. Determination of Sugars

Sugars analysis was performed only to ten samples of PDO honey (thyme-pine blend) due to insufficient quantities of other samples. Minor modifications were applied in the method of analysis with LC-RI of the Harmonized Methods of the International Honey Commission [18]: 3 g of honey was diluted to 100.0 mL of ACN : H_2_O (1 : 1 v/v). After filtration, 20 *μ*L aliquots were injected into the HPLC chromatograph with column Lichrospher 100-NH2, 250 × 4 mm i.d., 5 *μ*m, precolumn Lichrospher 100-NH2, 5 *μ*m, and refractive index detector. Isocratic elution was achieved by using ACN : H_2_O 80–20 (v/v) at 1.3 mL/min flow rate. Standard solution of sugars was prepared by dissolving 1.60 g of fructose, 1.50 g of glucose, and 0.3 g of sucrose in 100.0 mL ACN : H_2_O (1 : 1 v/v).

### 2.5. Statistical Analysis

Statistical data analysis was performed using the IBM SPSS software. One-way analysis of variance (ANOVA) was carried out to test the effect of one or several independent variables that defined groups of cases (botanical groups of honey samples) on the mean values of dependent variables. When a factor proved to cause significant differences (*P* < 0.05) in the mean of a dependent variable, Duncan's multiple range test (post hoc test) was applied in order to detect between which groups of cases differences occurred. The interactions between different dependent variables on the mean value of the dependent ones were investigated as well.

Multivariate statistical analysis was applied using the canonical discriminant analysis and Pearson's correlation analysis (proximity matrix produced for similarities between variables).

## 3. Results and Discussion

### 3.1. Determination of Physicochemical Parameters

The physicochemical parameters (botanical origin, color, water content, electrical conductivity, diastase activity, and hydroxymethylfurfural) of all samples of honey examined are shown in Table 1, and results (mean value, standard deviation, median, minimum, and maximum values) are summarized in Table 2. From the results, it is obvious that all samples examined are within the permitted limits for honey and safe in terms of authenticity [21, 22].

As expected according to the literature, pine and PDO thyme-pine honeys show darker colors (average value: 85.3 ± 8.4 mm and 78 ± 6.1 mm Pfund grades, Table 2, respectively), while Citrus honeys had the lowest Pfund grades (average value: 30.7 ± 4.5 mm). Citrus honeys showed higher color values from other Citrus honeys reported in the literature by Persano Oddo et al. [23] (15.0 ± 6.6 mm Pfund), Castiglioni et al. [24] (11 ± 5 mm Pfund), and Sant'Ana et al. [25] (20.06 mm Pfund).

The Pfund values for thyme honey (61.4 ± 16.0 mm Pfund, Table 2) are close to the ones reported for average European thyme honey (53.1 ± 10.8 mm) [13] and thyme honeys from Spain (80 ± 1.7 mm) [26] and from New Zealand (range 47–84 mm Pfund) [26].

Moreover, according to a study focused on Greek honey samples [27], it was reported that Greek thyme honey samples showed Pfund grades within the range 35–85 mm which complies with our results. Higher Pfund values indicate higher content in phenolic compounds and flavonoids [28].

### 3.2. Determination of Sugars

Results for the determination of fructose, glucose, and sucrose in PDO thyme-pine honeys are shown in Table 3. According to El Sohaimy et al. [29], the sugar composition of honey is affected by the type of flowers used by the bees, as well as climate conditions. All samples contained sucrose below 3% and total fructose + glucose higher than 50%, exactly as in the description of this PDO product [21].

The average ratio of fructose to glucose for the honey samples analyzed was found equal to 1.6 ± 0.1 (*n* = 10). This ratio depends largely on the source of the nectar from which the honey was extracted and allows evaluation of the crystallized glucose solubility levels in water as compared to fructose [20, 30, 31]. The amount of sucrose provides information about the maturity of honey as well as improper manipulation. High levels of sucrose indicate possible adulteration of honey [20, 21, 32].

### 3.3. Determination of Hydrogen Peroxide

Results for hydrogen peroxide in the honey samples examined are shown in Table 1, and results (mean value, standard deviation, median, minimum, and maximum values) are summarized in Table 2. Results are in accordance with other studies [20, 33, 34]. Among the four different botanical groups, the average hydrogen peroxide concentration is in the order thyme > PDO-thyme-pine = pine≈Citrus but no significant differences were observed.

### 3.4. Statistical Evaluation of Results

The correlation coefficient of Pfund values and electrical conductivity were found equal to 0.94 (*n* = 12) for thyme, 0.91 (*n* = 12) for PDO thyme-pine honey, and 0.95 (*n* = 6) for pine and Citrus honeys. Thus, it is confirmed that electrical conductivity is strongly associated with honey color which is in accordance with other studies [35]. Furthermore, the correlation coefficients of the concentration of glucose with Pfund grades and electrical conductivity of PDO thyme-pine honey were found equal to −0.74 and −0.79 (*n* = 10), respectively. Therefore, as the concentration of glucose increases, Pfund grades and electrical conductivity decrease with acceptable correlation.

**Figure 1:**
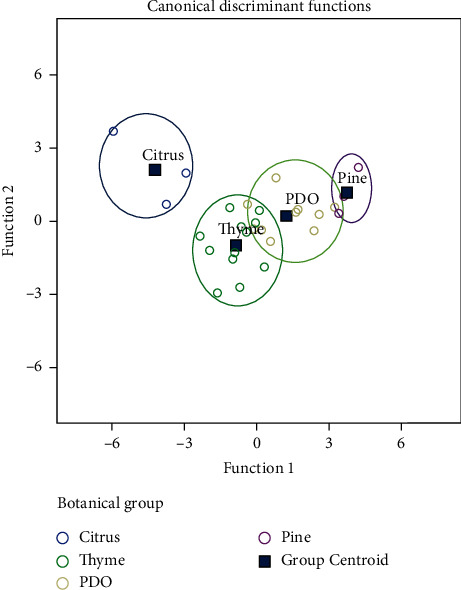
Canonical discriminant analysis of all honey samples examined.

All physicochemical parameters (except from sugars concentration) and concentration of hydrogen peroxide of all honey samples examined have been correlated by canonical discriminant analysis, which showed that 86.7% of the original grouped cases were correctly classified into the 4 botanical groups (Figure 1).

## 4. Conclusions

The present study showed that all honey samples from Crete produced hydrogen peroxide which plays an important role in the antibacterial activity of honey. Among the four different botanical groups, the average hydrogen peroxide concentration is in the order thyme > PDO-thyme-pine = pine≈Citrus, but no significant differences were observed. All physicochemical parameters (diastase activity, HMF content, moisture, electrical conductivity, color, and sugars) measured are in accordance with results of honeys from other countries [23–25].

Furthermore, univariate and multivariate statistics that have been applied to the analytical results have shown that a combination of the studied parameters can be also used to discriminate successfully honey sample groups of different botanical origins.

## Figures and Tables

**Table 1:** Results for color (Pfund grades), moisture content (*W*), electrical conductivity (EC), diastase activity (DN), hydroxymethylfurfural (HMF), and hydrogen peroxide (mM in 30% v/v aqueous honey solution) from honeys of different botanical origins.

Sample code	Pfund grades (mm)	*W* (g/100 g)	EC (mS/cm)	DN (Schade units)	HMF (mg/kg)	Hydrogen peroxide (mM) (mean ± SD, *n* = 3)
*Thyme honey*						
T1	71	14.6	0.52	9.0	13.9	0.033 ± 0.004
T2	43	14.6	0.33	23.2	4.3	0.075 ± 0.013
T3	33	14.6	0.27	26.1	5.8	0.092 ± 0.003
T4	53	15.4	0.39	31.9	2.4	0.028 ± 0.007
T5	56	15.6	0.47	43.6	4.9	0.077 ± 0.002
T6	68	15.4	0.50	27.8	8.3	0.028 ± 0.005
T7	74	14.2	0.52	21.1	9.0	0.032 ± 0.007
T8	84	16.0	0.63	25.4	8.1	0.028 ± 0.005
T9	84	14.5	0.59	27.1	12.6	0.032 ± 0.003
T10	56	14.3	0.51	16.5	11.1	0.035 ± 0.005
T11	47	14.3	0.43	8.5	3.7	0.034 ± 0.005
T12	68	14.8	0.54	10.9	4.6	0.043 ± 0.004

*PDO “Pefkothymaromelo Kritis” (blend thyme-pine) honey*						
PDO-1	76	14.4	0.74	9.2	4.5	0.041 ± 0.006
PDO-2	70	14.5	0.69	9.7	3.0	0.035 ± 0.005
PDO-3	74	14.7	0.65	9.5	11.5	0.034 ± 0.006
PDO-4	87	13.4	0.89	8.5	9.9	0.036 ± 0.006
PDO-5	80	14.0	0.95	13.3	4.5	0.048 ± 0.006
PDO-6	73	14.3	0.65	17.8	6.6	0.049 ± 0.009
PDO-7	72	14.6	0.66	7.3	2.8	0.031 ± 0.006
PDO-8	75	15.8	0.80	14.2	4.9	0.027 ± 0.005
PDO-9	76	14.7	0.68	18.0	11.0	0.038 ± 0.006
PDO-10	80	14.4	0.81	8.5	4.2	0.039 ± 0.006
PDO-11	90	14.4	0.93	7.7	4.6	0.043 ± 0.006
PDO-12	83	13.8	0.87	7.3	5.4	0.042 ± 0.009

*Pine honeydew honey*						
P-1	80	14.5	1.16	5.2	1.2	0.030 ± 0.005
P-2	81	13.8	0.96	8.2	5.2	0.028 ± 0.006
P-3	95	15.2	1.11	22.7	1.9	0.050 ± 0.007

*Orange blossom (Citrus) honey*						
C-1	35	16	0.19	15	36.9	0.010 ± 0.002
C-2	31	15.2	0.20	11.4	7.8	0.054 ± 0.009
C-3	26	16.4	0.45	22.8	12.8	0.050 ± 0.004

**Table 2:** Variation of color (Pfund grades), moisture content (*W*), electrical conductivity (EC), diastase activity (DN), hydroxymethylfurfural (HMF), and hydrogen peroxide (mM in 30% v/v aqueous honey solution) among all honeys examined.

	Mean	±SD (*n*)	Median	Min	Max
*Thyme honey (n* *=* *12)*					
Pfund grades (mm)	61.4	16.0	62.0	33.0	84.0
*W* (g/100 g)	14.9	0.59	14.6	14.2	16.0
EC (mS/cm)	0.48	0.10	0.50	0.27	0.63
DN	22.6	10.2	24.3	8.5	43.6
HMF (mg/kg)	7.4	3.7	7.0	2.4	13.9
H_2_O_2_ (mM)	0.045	0.022	0.034	0.028	0.092

*PDO “Pefkothymaromelo Kritis” (blend thyme-pine) honey (n* *=* *12)*					
Pfund grades (mm)	78.0	6.1	76.0	70.0	90.0
*W* (g/100 g)	14.4	0.6	14.4	13.4	15.8
EC (mS/cm)	0.77	0.10	0.77	0.65	0.91
DN	10.9	3.9	9.4	7.3	18.0
HMF (mg/Kg)	6.1	3.0	4.8	2.8	11.5
H_2_O_2_ (mM)	0.038	0.006	0.038	0.027	0.049

*Pine honeydew honey (n* *=* *3)*					
Pfund grades (mm)	85.3	8.4	81.0	80.0	95.0
*W* (g/100 g)	14.5	0.7	14.5	13.8	15.2
EC (mS/cm)	1.08	0.10	1.11	0.96	1.16
DN	12.1	9.4	8.2	5.1	22.7
HMF (mg/kg)	2.8	2.1	1.9	1.2	5.2
H_2_O_2_ (mM)	0.036	0.012	0.030	0.028	0.050

*Orange blossom (Citrus) honey (n* *=* *3)*					
Pfund grades (mm)	30.7	4.5	31.0	26.0	35.0
*W* (g/100 g)	15.9	0.6	16.0	15.2	16.5
EC (mS/cm)	0.26	0.12	0.20	0.19	0.46
DN	16.4	5.9	15.0	11.4	22.8
HMF (mg/kg)	19.1	15.6	12.8	7.78	36.9
H_2_O_2_ (mM)	0.038	0.024	0.050	0.010	0.054

**Table 3:** Results for the determination of fructose, glucose, and sucrose in PDO “Pefkothymaromelo Kritis” (blend thyme-pine honey) (*n* = 10).

Sample code	Fructose (g/100 g)	Glucose (g/100 g)	Fructose + glucose (g/100 g)	Sucrose (g/100 g)	Ratio [fructose]/[glucose]
PDO-1	35.7	23.3	59.0	n.d.	1.5
PDO-2	34.8	22.9	57.8	n.d.	1.5
PDO-3	39.0	24.6	63.6	0.7	1.6
PDO-4	33.4	20.7	54.1	2.2	1.6
PDO-5	35.0	20.8	55.8	n.d.	1.7
PDO-6	35.5	25.6	61.1	2.4	1.4
PDO-7	34.0	22.3	56.3	1.3	1.5
PDO-8	33.8	24.0	57.9	n.d.	1.4
PDO-10	33.9	20.5	54.4	n.d.	1.7
PDO-11	33.6	20.3	53.8	n.d.	1.7
Mean value					
±SD (*n* = 10)	34.9 ± 1.7	22.5 ± 1.9	57.4 ± 3.2	0.7 ± 1.0	1.6 ± 0.1

n.d.: not detected.

## Data Availability

The data used to support the findings of this study are available from the corresponding author.
